# Olfactory Bulb and Gyrus Rectus Volumes in Alzheimer’s Disease: Associations with Eating Disturbances

**DOI:** 10.3390/medicina62081615

**Published:** 2026-08-21

**Authors:** Feride Fatma Görgülü, Orhan Görgülü

**Affiliations:** 1 Department of Radiology, Adana Health Practice and Research Center, University of Health Sciences, Adana 01230, Türkiye; 2 Department of Otorhinolaryngology, Adana Health Practice and Research Center, University of Health Sciences, Adana 01330, Türkiye

**Keywords:** Alzheimer’s disease, olfactory bulb, gyrus rectus, MRI volumetry, eating disturbances, neurodegeneration

## Abstract

*Background and Objectives*: Olfactory dysfunction is an early non-cognitive feature of Alzheimer’s disease (AD). The volumetric behavior of olfactory and related frontal structures and their link to eating disturbances remains unclear; we therefore compared olfactory bulb (OB) and gyrus rectus (GR) volumes between patients with AD and controls and examined their relationship with eating disturbances and their diagnostic value. *Materials and Methods*: In this single-center, retrospective, case–control study, 135 patients with AD and 49 age-matched controls underwent 3-Tesla MRI. Right, left, and total OB and GR volumes were measured. Groups were compared using the Mann–Whitney U test; age- and sex-adjusted logistic regression and receiver operating characteristic (ROC) analyses were performed; and OB and GR volumes were compared according to eating disturbance status in patients with AD, with adjustment for age and sex. *p*-Values were corrected for multiple comparisons using the Benjamini–Hochberg false discovery rate procedure. *Results*: All OB and GR volumes were significantly lower in patients with AD compared to controls (all *p* < 0.001), with the total OB volume nearly half that of the controls. Among patients with AD, all OB volumes were significantly lower in those with eating disturbances (all *p* < 0.001) and remained independently associated after adjustment for age and sex, whereas none of the GR volumes was significantly associated with eating disturbance status after adjustment. In adjusted models, every volumetric measure was independently associated with AD (all *p* < 0.001). The total OB volume showed the best diagnostic performance (AUC 0.940; sensitivity 0.82; specificity 0.98), while GR volumes performed less well (AUC 0.78–0.84). *Conclusions*: AD is associated with marked OB and GR atrophy. OB volume was associated with eating disturbances and discriminated AD from controls with high accuracy, supporting OB volumetry as an accessible candidate imaging marker that warrants prospective validation.

## 1. Introduction

Alzheimer’s disease (AD) is a progressive degenerative disorder of the central nervous system characterized by cognitive decline, memory impairment, and a gradual loss of functional ability [1]. As the leading cause of dementia worldwide, AD places a heavy clinical, social, and economic burden on society [2]. The classical pathological features of AD, including amyloid-β plaques, neurofibrillary tangles, synaptic loss, and cortical atrophy, are well described. In recent years, however, growing attention has been paid to early non-cognitive changes that may precede overt cognitive impairment [3]. Among these, olfactory dysfunction has emerged as one of the most reliable early signs of AD-related neurodegeneration, and olfactory decline has been shown to predict the conversion from mild cognitive impairment (MCI) to AD [4,5].

Olfactory impairment in AD has been linked to structural and functional changes along the olfactory pathway, including to the olfactory epithelium, olfactory bulb (OB), olfactory tract, and the central processing regions of the limbic and frontal lobes [6]. The OB is particularly vulnerable in neurodegenerative disease. Reduced OB volume has been reported in several neurological and psychiatric conditions, such as Parkinson’s disease, major depressive disorder, traumatic olfactory dysfunction, sinonasal disease, and post-infectious olfactory dysfunction [7,8,9]. Because the human OB retains some structural plasticity and responds to both peripheral and central influences, a smaller OB volume is thought to reflect reduced afferent sensory input and lower neurogenesis [10]. In AD, however, the few existing volumetric studies have yielded inconsistent results, with some reporting clear OB atrophy and others finding no significant difference from controls [11,12]. The volumetric behavior of the OB in AD, and its relationship with associated behavioral disturbances, therefore remains far less well characterized than in other conditions.

The gyrus rectus (GR) is a frontal lobe structure of particular interest for olfactory processing. Although it is often regarded as a primitive or functionally simple region, the GR is closely connected to key components of the olfactory and limbic systems and contributes to emotional regulation, reward processing, and aspects of executive function [13]. Reduced GR volume has been described in depression and other neuropsychiatric disorders, which suggests that this structure is sensitive to neurodegenerative and neurofunctional change [14]. Given its proximity to the OB and its links to cognitive–emotional circuits, GR volumetry may help clarify the structural basis of olfactory and behavioral impairment in AD.

A further under-recognized feature of AD is disturbed eating behavior. Patients often show altered appetite and food preferences, reduced hunger signaling, and neglect of normal eating routines [15]. These disturbances can lead to malnutrition, weight loss, increased morbidity, and reduced quality of life. Because olfaction plays a central role in appetite, flavor perception, and the hedonic response to food, impaired smell may directly affect eating behavior [16]. Despite this plausible link, few studies have examined whether structural changes in olfactory-related brain regions are associated with eating disturbances in AD.

Overall, current evidence indicates that OB atrophy is a common consequence of neurodegenerative and neuropsychiatric disease, while GR changes may accompany disorders involving emotional and behavioral symptoms [6,17,18]. These findings point to a possible interaction between the olfactory system, the frontal lobe, and behavioral expression that has not been fully explored in AD. The present study addresses these gaps using a retrospective, case–control design to compare OB and GR volumes between patients with AD and healthy controls using standardized volumetric measurement. We further examined the correlations among OB and GR volumes, assessed their ability to discriminate patients with AD from controls, and explored whether OB and GR volumes differ according to the presence of eating disturbances in patients with AD. By combining structural, behavioral, and clinical data, this study aims to clarify the neuroanatomical basis of olfactory dysfunction and disordered eating in Alzheimer’s disease.

## 2. Materials and Methods

### 2.1. Study Design

This was a single-center, retrospective, case–control study designed to compare the volumes of the OB and GR between patients with AD and healthy controls. Clinical and imaging data were retrieved from the hospital archive, which allowed a relatively large cohort to be analyzed under routine clinical conditions and thereby strengthened the ecological validity of the findings. The study was conducted in accordance with the Declaration of Helsinki and was approved by the Clinical Research Ethics Committee of Adana City Hospital (No.: 408/13-03-2019, issued on 13 March 2019). Due to the retrospective design, the requirement for written informed consent was waived.

### 2.2. Study Population

The study population comprised 135 patients with AD and 49 age-matched controls, for a total of 184 participants.

Controls were individuals who had undergone brain MRI for non-neurological indications, such as examination of possible structures compressing the optic nerve, evaluation of inner ear structures and auditory nerves due to complaints of hearing loss and tinnitus, and whose imaging results were reported as normal. None of the controls had a history of neurological disease, psychiatric illness, or cognitive impairment. Controls were selected so as to match the age distribution of the AD group.

### 2.3. Inclusion and Exclusion Criteria

Patients were included in the AD group if they had a documented diagnosis of Alzheimer’s disease, were aged 50 years or older, and had complete MRI data suitable for volumetric analysis. All patients were diagnosed by an experienced neurologists using the Diagnostic and Statistical Manual of Mental Disorders (DSM-5) criteria and the International Classification of Diseases (ICD-11) systems. Most patients underwent cognitive function tests, but only some had biomarker confirmation. For both groups, the exclusion criteria were a history of major head trauma, intracranial tumor or mass lesion, previous brain surgery, severe motion artifact degrading image quality, and any systemic or neurological disorder known to affect olfactory processing. Mixed dementia cases that did also not meet the criteria were excluded. Applying these criteria helped ensure that the observed volumetric differences were attributable primarily to AD-related pathology rather than to confounding structural or neurological abnormalities.

### 2.4. MRI Acquisition and Volumetric Measurement

All MRI examinations were performed using a 3-Tesla unit scanner with a standardized imaging protocol optimized for the visualization of the olfactory structures and frontal cortex. All images were evaluated using the Philips IntelliSpace workstation (Philips Ingenia, Best, Eindhoven, The Netherlands, 2017). The sequence parameters were as follows: axial T1-weighted [repetition time (TR): 550–750 ms; echo time (TE): 20–25 ms; scan thickness: 3 mm; slice gap: 1 mm; and matrix: 256 × 256], axial T2-weighted (TR: 4000–5000 ms; TE: 90–120 ms; scan thickness: 3 mm; slice gap: 1 mm; and matrix: 256 × 256), sagittal T2-weighted fluid-attenuated inversion recovery (FLAIR) (TR: 7200 ms; TE: 120 ms; FA: 90°; TI: 1333–2041 ms; and matrix, 256 × 256), and coronal T2 (TR: 6550 ms; TE: 99 ms, flip angle: 150°; slice thickness: 3 mm; and matrix: 256 × 256).

**Figure 1:**
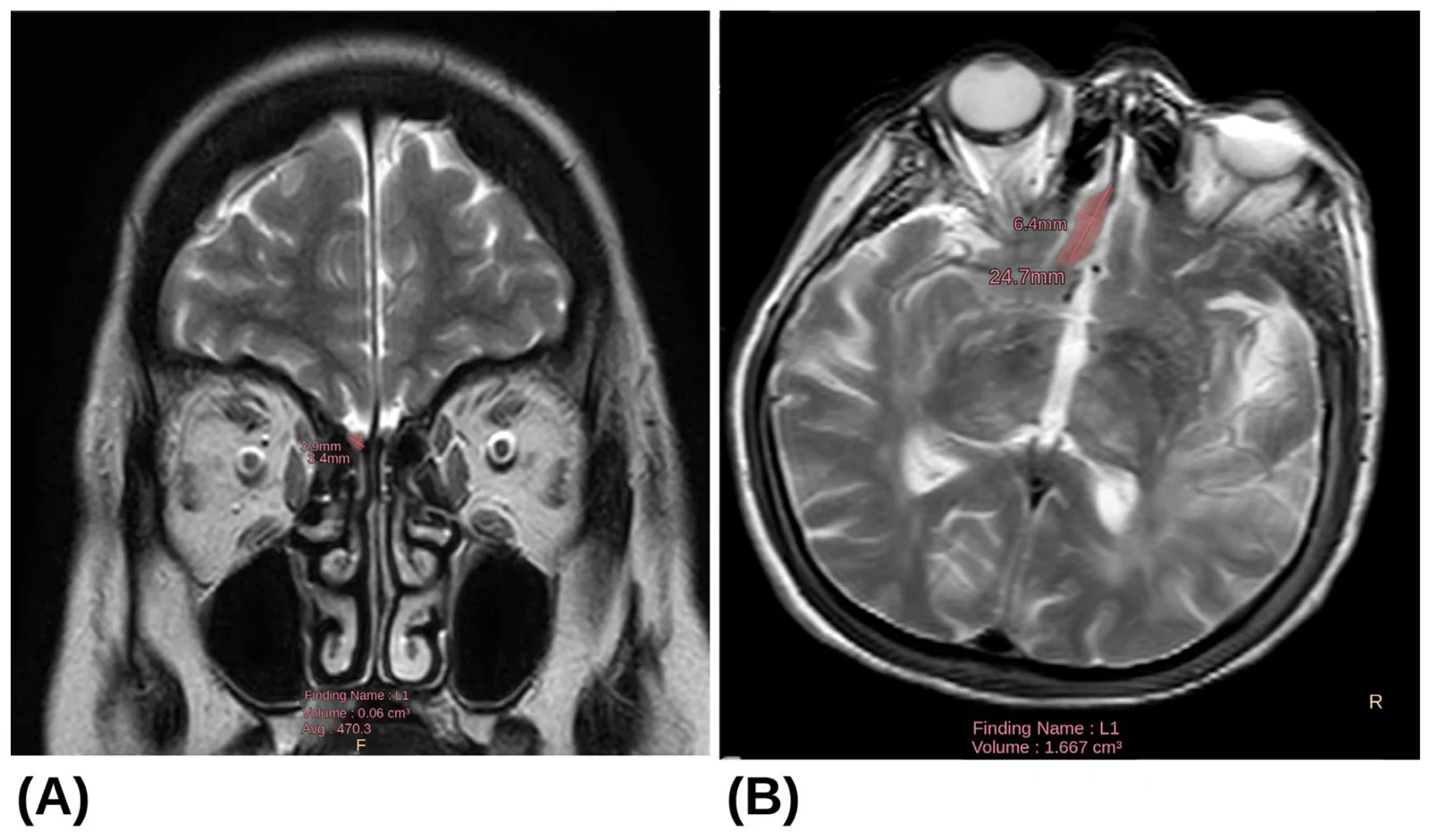
MRI-based volumetric assessment of the olfactory bulb and gyrus rectus. (**A**) Measurement of olfactory bulb volume on a T2-weighted coronal MRI image. (**B**) Measurement of gyrus rectus volume on a T2-weighted axial MRI image.

The OB volumes were measured on T2-weighted coronal images, which provide high soft-tissue contrast for precise delineation of the olfactory bulbs. The GR volumes were assessed on axial images, allowing optimal evaluation of frontal lobe morphology. Representative MRI images demonstrating OB and GR measurements are presented in Figure 1A and Figure 1B, respectively.

All volumetric measurements were performed by a single radiologist with 15 years of experience, who was blinded to group allocation. Volumes were obtained by manual segmentation using a tumor-tracking-based segmentation tool the Philips IntelliSpace workstation, a technique well suited to delineating small anatomical structures and yielding reproducible volumetric estimates. The right and left OB and GR volumes were measured three times each on separate days to eliminate the memory effect and ensure data independence. The average of these three values was taken as the value. Total volumes were calculated as the sum of the bilateral values.

### 2.5. Assessment of Eating Disturbances

Eating disturbance status in patients with AD was determined from clinical notes and assessments recorded in the hospital electronic medical record. Patients were classified into two groups: those with documented eating disturbances, including loss of appetite, altered food preferences, overeating, or abnormal meal timing, and those without any such symptoms. This dichotomous classification enabled subgroup comparison of the volumetric measurements.

### 2.6. Statistical Analysis

The primary outcome was the discriminative performance of total olfactory bulb volume for distinguishing patients with AD from controls; gyrus rectus measures, eating disturbance subgroup comparisons, and correlation analyses were considered secondary and exploratory. Data were analyzed using non-parametric and categorical methods. Continuous variables were expressed as median and interquartile range [Q1–Q3], while categorical variables were reported as frequencies and percentages. The distribution of continuous variables was checked before any group comparison. Because the volumetric MRI measurements were not normally distributed, two independent groups were compared using the Mann–Whitney U test, with effect sizes reported as rank-biserial correlations with 95% confidence intervals. To account for multiple comparisons, *p*-values were adjusted using the Benjamini–Hochberg FDR procedure, applied separately within each family of related tests. FDR was preferred over Bonferroni because the correlated volumetric measures make the latter unduly conservative. Adjusted *p*-values (q-values) are reported alongside unadjusted values, with significance defined as FDR-adjusted *p* < 0.05. Categorical variables were compared with the chi-square test or Fisher’s exact test, whichever was appropriate. Spearman’s rank correlation was used to assess the relationships between age and the MRI volumetric measurements. To examine the association between these measurements and Alzheimer’s disease, separate logistic regression models were built. In each model, AD status served as the dependent variable, and a single MRI volumetric variable was entered as the predictor after adjusting for age and gender. The same age- and sex-adjusted logistic regression approach was applied within the AD group to compare volumetric measurements according to eating disturbance status. All volumetric variables were standardized before regression; therefore, the odds ratios reflect the change in the odds of AD for each one-standard-deviation increase in the corresponding measurement. Multicollinearity was checked using the variance inflation factor (VIF) and tolerance values. Finally, receiver operating characteristic (ROC) curve analysis was carried out to assess how well the MRI volumetric measurements distinguished patients with AD from controls. For each measurement, the area under the curve (AUC), 95% confidence interval, optimal cut-off value, sensitivity, and specificity were calculated. A *p*-value below 0.05 was accepted as statistically significant. All statistical analyses and graphical outputs were produced in R (R Core Team, Vienna, Austria; https://www.R-project.org/, accessed on 21 August 2026) using the RStudio integrated development environment (Posit PBC, Boston, MA, USA; https://posit.co/, accessed on 21 August 2026).

## 3. Results

### 3.1. Descriptive Characteristics of the Study Population

The study included 184 participants, which consisted of 135 patients with AD and 49 controls. The median age was 78 [71–81.5] years in the AD group and 74 [71–80] years in the control group, with no significant difference between them (*p* = 0.262). Gender distribution was also similar, as females made up 69.6% of the AD group and 67.3% of the control group (*p* = 0.907). Eating disturbance status, however, differed significantly. It was present in 65.2% of patients with AD but in none of the controls (*p* < 0.001) (Table 1).

### 3.2. Comparison of MRI Volumetric Measurements Between Groups

All olfactory bulb and gyrus rectus volumes were significantly lower in patients with AD than in controls (all FDR-adjusted *p* < 0.001; Table 2). The difference was most pronounced for the olfactory bulb, where the total volume in patients was roughly half that of controls (0.079 versus 0.139) and the effect sizes were large (rank-biserial r = 0.85–0.89). The gyrus rectus volumes showed a similar but more moderate reduction (r = 0.56–0.68).

**Figure 2:**
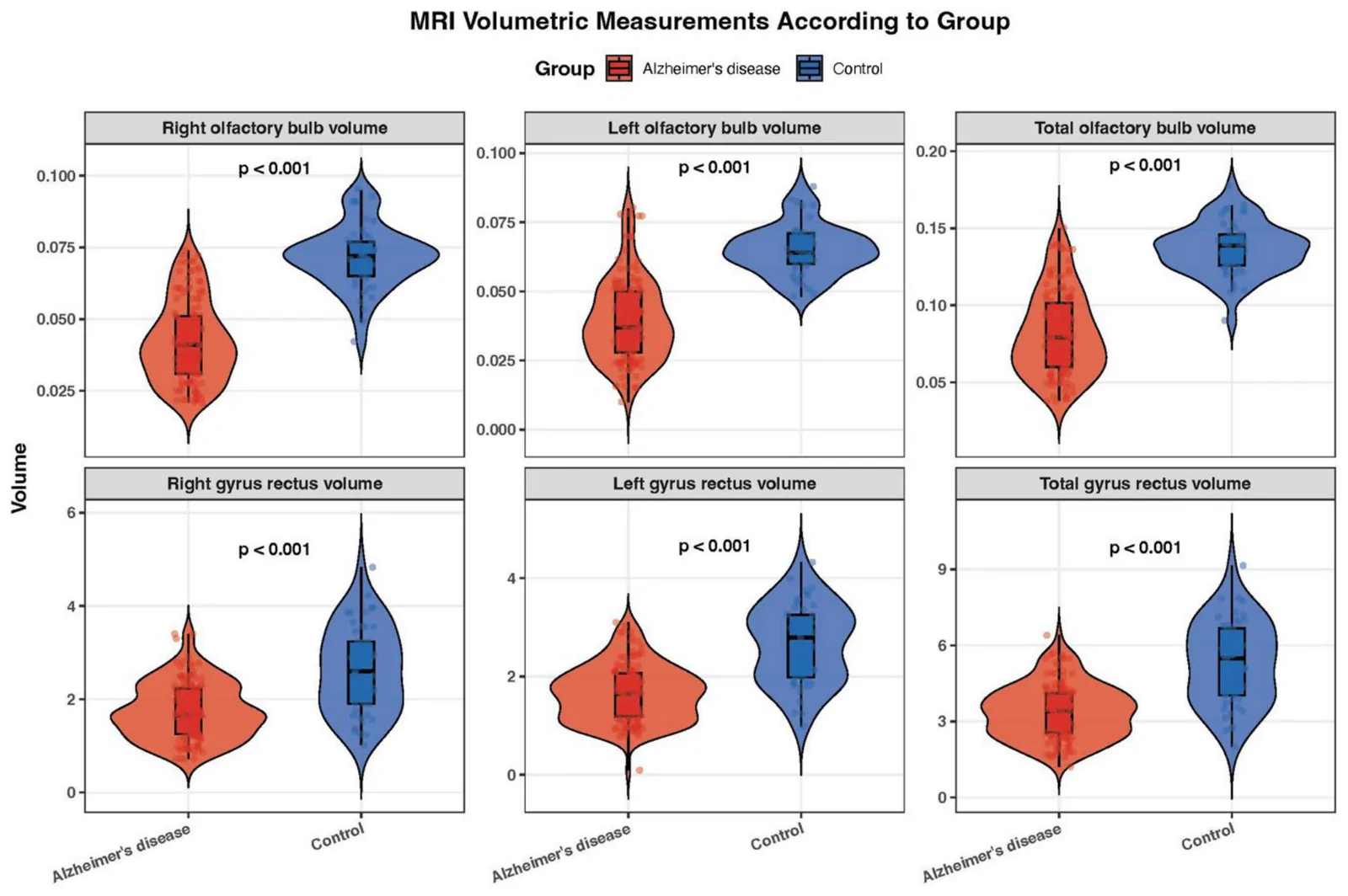
Violin plots with embedded boxplots show the distribution of right, left, and total olfactory bulb volumes and right, left, and total gyrus rectus volumes in patients with Alzheimer’s disease and controls. Boxes represent the interquartile range, horizontal lines indicate the median, whiskers show the data range, and individual points represent participant-level values. Group comparisons were performed using the Mann–Whitney U test. OB: olfactory bulb; GR: gyrus rectus. *p* < 0.001 was considered statistically significant.

These differences remained significant after adjustment for age and sex, with every volume independently associated with AD (adjusted OR 0.08–0.27, all *p* < 0.001; Table 2). These patterns are also illustrated by the violin plots, which show consistently smaller olfactory bulb and gyrus rectus volumes in the AD group across all parameters (see Figure 2).

### 3.3. MRI Volumetric Measurements According to Eating Disturbance Status in Patients with Alzheimer’s Disease

**Figure 3:**
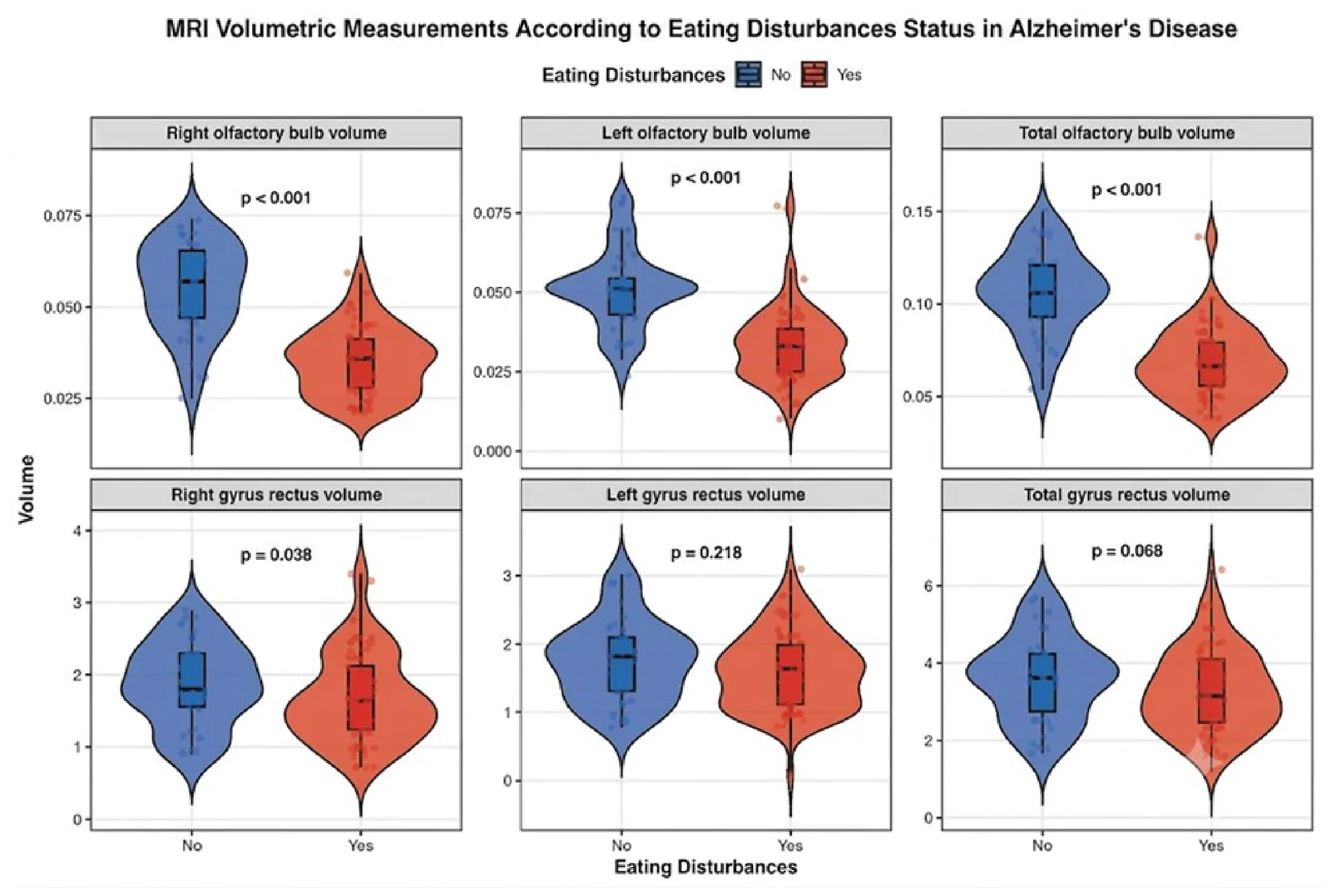
Violin plots with embedded boxplots show the distribution of right, left, and total olfactory bulb volumes and right, left, and total gyrus rectus volumes in Alzheimer’s disease patients with and without eating disturbances. Boxes represent the interquartile range, horizontal lines indicate the median, whiskers show the data range, and individual points represent participant-level values. Group comparisons were performed using the Mann–Whitney U test. OB: olfactory bulb; GR: gyrus rectus. *p* < 0.05 was considered statistically significant.

Among patients with Alzheimer’s disease, all three olfactory bulb volumes were significantly lower in those with eating disturbances than in those without (all FDR-adjusted *p* < 0.001). In unadjusted comparisons, the right gyrus rectus volume also differed (*p* = 0.038), whereas the left and total gyrus rectus volumes did not (see Figure 3).

After adjustment for age and sex in multivariable logistic regression (Table 3), all olfactory bulb volumes remained independently associated with eating disturbance status (adjusted OR 0.06–0.13, all *p* < 0.001), whereas none of the gyrus rectus volumes was significantly associated, including the right side (adjusted OR 0.80, 95% CI 0.45–1.44, *p* = 0.45).

### 3.4. Correlation Analysis

**Figure 4:**
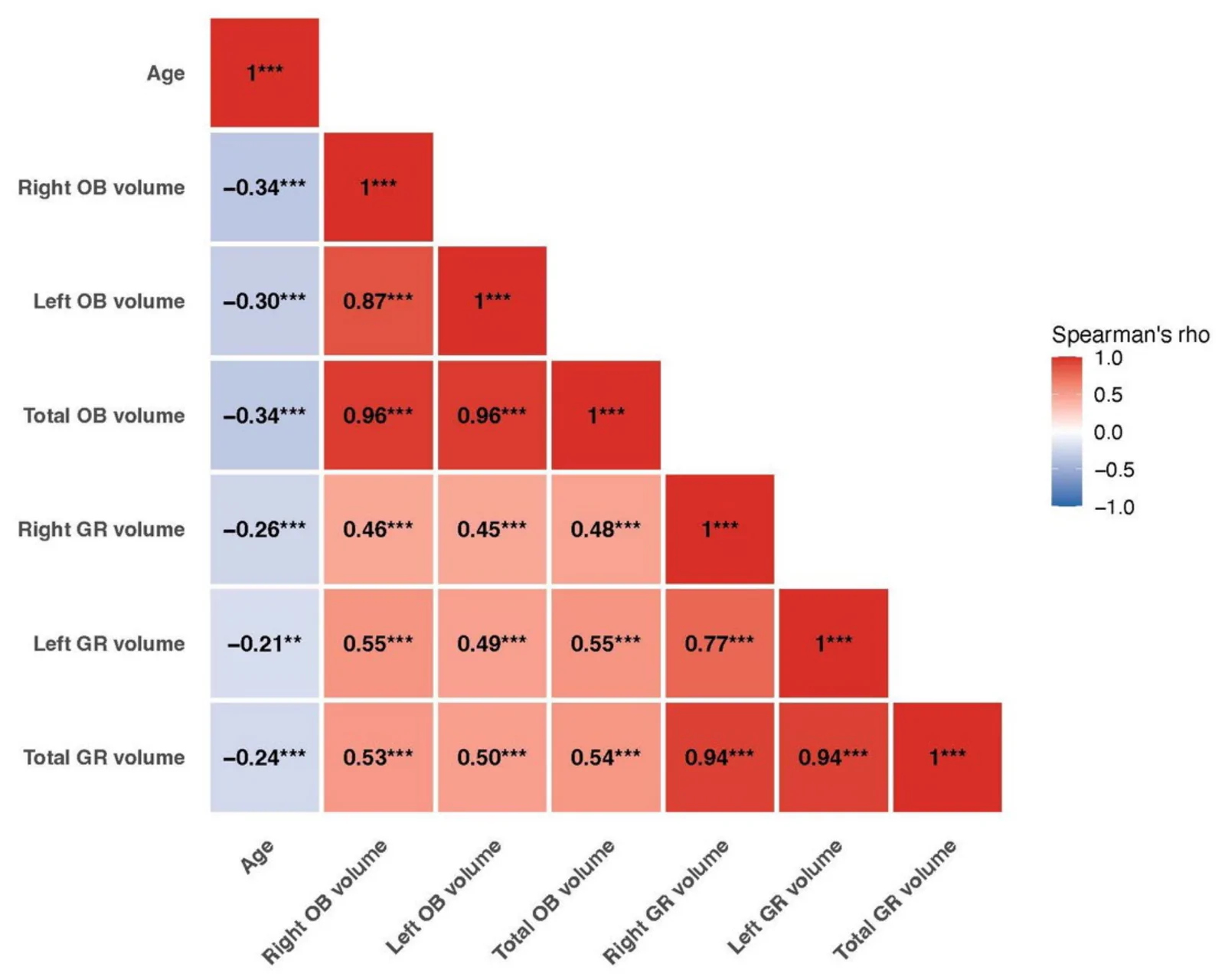
Spearman correlation matrix of age and MRI volumetric measurements. Values indicate Spearman’s rho coefficients. Asterisks indicate statistical significance: ** *p* < 0.01, *** *p* < 0.001. OB: olfactory bulb; GR: gyrus rectus.

Spearman’s correlation analysis revealed significant negative correlations between age and the MRI volumetric measurements. Age was inversely correlated with the right, left, and total olfactory bulb volumes, as well as with the right, left, and total gyrus rectus volumes. The strongest negative correlations with age were found for the right olfactory bulb volume and the total olfactory bulb volume, both with a rho of −0.34. The olfactory bulb volumes were also strongly intercorrelated. The right and left volumes correlated closely with each other and with the total olfactory bulb volume. The same was true for the gyrus rectus volumes, where the right and left measurements correlated strongly with the total. In addition, positive correlations were seen between the olfactory bulb and gyrus rectus measurements, suggesting that lower olfactory bulb volumes tended to occur together with lower gyrus rectus volumes (see Figure 4).

### 3.5. Logistic Regression Analysis

In the age- and sex-adjusted logistic regression models, every MRI volumetric measurement was significantly associated with AD status. Larger olfactory bulb volumes were associated with lower odds of AD (adjusted OR 0.089 right, 0.127 left, 0.083 total; all *p* < 0.001), as were larger gyrus rectus volumes (adjusted OR 0.265 right, 0.184 left, 0.196 total; all *p* < 0.001), with no problematic multicollinearity (all VIF < 1.2) (see Table 4).

### 3.6. Nested Model Comparison and Incremental Predictive Value

Because the olfactory bulb and gyrus rectus are anatomically related and volumetrically correlated, we tested whether each structure contributed independent, incremental information for discriminating patients with AD from controls. Nested age- and sex-adjusted logistic regression models were constructed using total OB and total GR volumes as representative volumetric measures (Table 5). The base model (age and sex) discriminated poorly (AUC = 0.554, 95% CI: 0.463–0.645). Adding total OB volume markedly improved model fit (likelihood-ratio test, *p* < 0.001), lowered the AIC from 218.9 to 86.3, and raised the AUC to 0.963 (95% CI: 0.939–0.987; DeLong *p* < 0.001 vs. base model). Subsequent addition of total GR volume produced a further significant improvement (likelihood-ratio test, *p* < 0.001; AIC = 74.8; AUC = 0.977, 95% CI: 0.960–0.995; DeLong *p* = 0.039 vs. OB model). In the combined model, both total OB volume (adjusted OR = 0.011, 95% CI: 0.001–0.052; *p* < 0.001) and total GR volume (adjusted OR = 0.284, 95% CI: 0.119–0.579; *p* = 0.002) remained independently associated with AD, with all variance inflation factors below 1.8, indicating no substantial multicollinearity.

### 3.7. ROC Analysis

**Figure 5:**
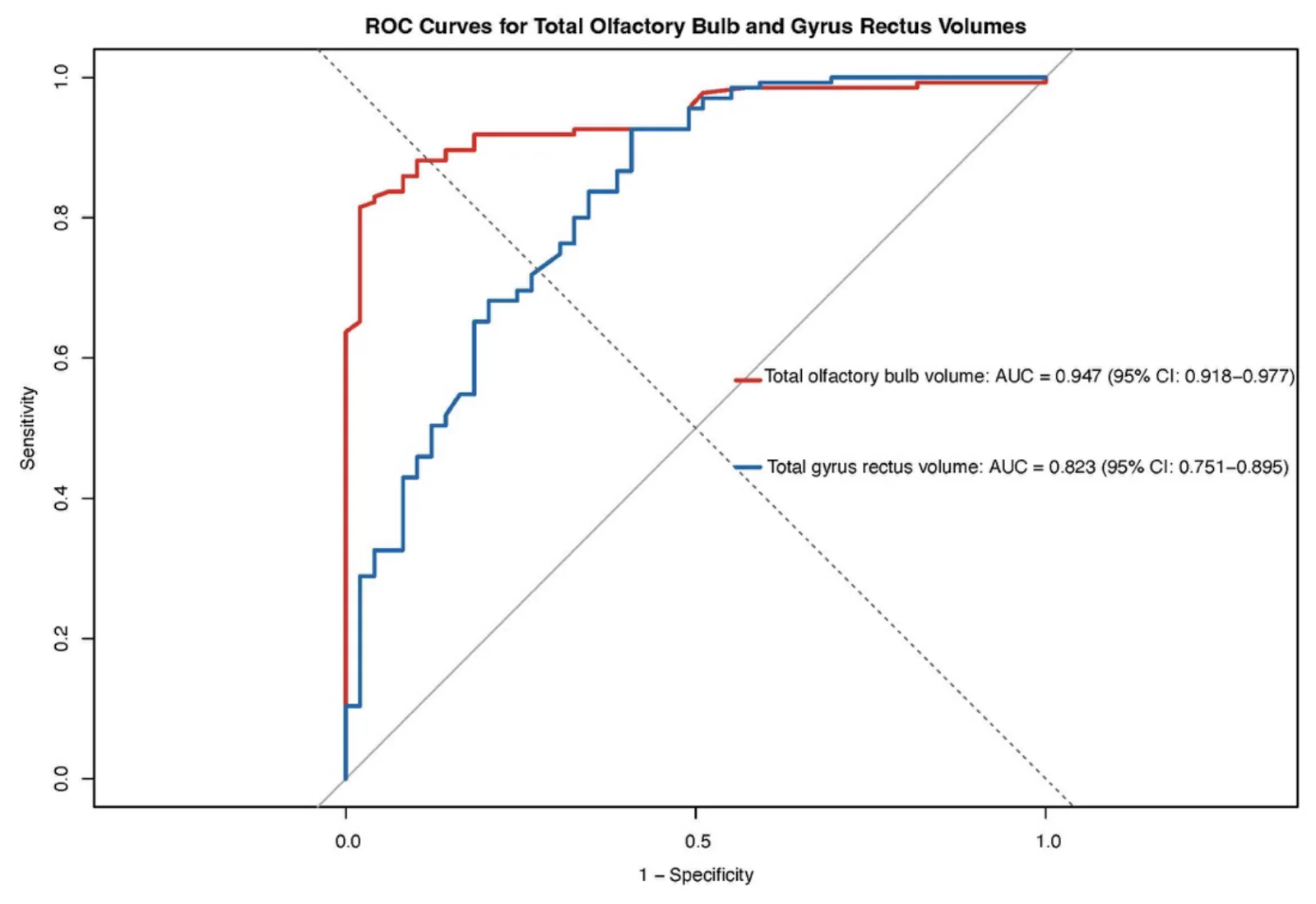
Receiver operating characteristic (ROC) curves show the diagnostic performance of total olfactory bulb volume and total gyrus rectus volume in discriminating patients with Alzheimer’s disease from controls. The solid grey diagonal line represents the line of no discrimination (AUC = 0.50), and the dotted grey line is the anti-diagonal, along which sensitivity equals specificity. AUC: area under the curve; CI: confidence interval.

The olfactory bulb volumes showed excellent diagnostic performance in discriminating patients with AD from controls, with the total olfactory bulb volume performing best (AUC 0.940; sensitivity 0.815; specificity 0.980). The gyrus rectus volumes had lower but still clinically useful discriminatory power (AUC range 0.778–0.840). Consistent with these results, the ROC curve comparison confirmed the superior diagnostic performance of the total olfactory bulb volume over the total gyrus rectus volume (see Figure 5).

## 4. Discussion

This study examined volumetric changes in the OB and GR in patients with AD compared with healthy controls, and explored whether these changes were related to eating disturbances in patients with AD. Both OB and GR volumes were significantly lower in patients with AD than in controls. Among patients with AD, all OB volumes were significantly smaller in those with eating disturbances, whereas for the GR this association was limited to the right side. In addition, OB volumetry showed an excellent ability to distinguish patients with AD from controls. Overall, these findings point to a possible structural basis for the olfactory dysfunction and behavioral changes seen in AD.

As a biological definition in current Alzheimer’s diagnosis, a positive Core-1 biomarker (such as abnormal amyloid PET or plasma p-tau217) is sufficient, even in people without cognitive symptoms. Additionally, core-1 markers establish if the disease is present, while clinical evaluation and secondary markers (like tau-PET or neurodegeneration metrics) evaluate disease stage. It is also now widely accepted that CSF Aβ42 (or the Aβ42/Aβ40 ratio) is a valid indicator of the abnormal pathologic state. In addition to these biomarkers, we attempted to find a candidate imaging marker [1].

The pronounced reduction in OB volume in patients with AD is consistent with the growing evidence that olfactory impairment is one of the earliest non-cognitive features of AD pathology. OB atrophy has been reported not only in AD but also in other neurodegenerative and psychiatric conditions, including Parkinson’s disease, depression, post-infectious olfactory loss, sinonasal disease, and idiopathic olfactory dysfunction [19]. These conditions share disrupted olfactory signaling, which is often linked to reduced peripheral input or impaired central processing [6]. In the present cohort, the total OB volume in patients was nearly half that of controls, corresponding to a reduction of approximately 43%. This finding is in line with earlier MRI studies that reported reduced olfactory bulb and tract volumes in early AD and in mild cognitive impairment (MCI), and with a meta-analysis showing smaller OB volumes in both AD and MCI, although with considerable heterogeneity across studies [5,12,20]. Because the OB is frequently one of the first structures affected by neurodegeneration, this degree of volume loss supports its inherent vulnerability and its potential value as an early imaging biomarker of AD-related neurodegeneration [21]. It should be noted, however, that not all studies have confirmed this pattern. Some have found no significant difference in OB volume between patients with AD or MCI and controls, particularly in the earliest stages, and have suggested that detectable OB atrophy may emerge only later in the disease course [11,22]. The robust group difference observed in our larger cohort may reflect a more advanced disease stage and the relatively wide age range of our patients.

The marked atrophy of the GR observed here highlights the involvement of frontal lobe structures in AD. The GR lies in the medial orbitofrontal cortex, close to the OB and to key limbic pathways [23]. Although it has traditionally been viewed as a functionally simple region, accumulating evidence suggests that the GR contributes to emotional processing, reward evaluation, decision making, and aspects of working memory. Reduced GR volume has previously been reported in depression and other neuropsychiatric disorders, which suggests that this structure is particularly sensitive to disturbances of affective and cognitive regulation [24]. Volume loss in the gyrus rectus and adjacent orbitofrontal cortex has also been described in dementia syndromes, and gray-matter loss in prefrontal and orbitofrontal regions has been linked to neuropsychiatric symptoms in AD [25,26]. At the same time, some studies indicate that frontal involvement in AD is less pronounced than in frontotemporal degeneration, so the marked GR atrophy seen here should be interpreted within this context [27]. Overall, the significant GR volume loss in our patients indicates that structural degeneration in frontal regions may be more widespread in AD than is often recognized.

A further finding was the consistent positive correlation between OB and GR volumes. This relationship probably reflects the close anatomical and functional links between olfactory and prefrontal regions within wider neural networks. The olfactory system projects directly to the orbitofrontal cortex and is one of the few sensory systems that reach the cortex without first relaying through the thalamus, which allows rapid integration of olfactory input with cognitive and emotional processing [28]. The parallel volume reductions in the OB and GR may therefore result from a shared neurodegenerative process, or from the secondary effects of reduced olfactory input on downstream frontal regions. Although these correlations were modest, they were consistent across measurements, which supports the idea of coordinated structural decline within olfactory–frontal networks.

The link between eating disturbances and volumetric measures is a novel aspect of this study. Eating disturbances such as loss of appetite, altered taste and food preferences, reduced enjoyment of eating, and changes in eating patterns are common in AD and contribute substantially to morbidity and functional decline. In our cohort, patients with AD who had eating disturbances showed significantly lower OB volumes than those without, which provides a possible neuroanatomical basis for these behavioral symptoms [29]. Olfaction plays a central role in appetite regulation, flavor perception, and the hedonic evaluation of food; a smaller OB volume may impair the perception of food-related odors, weaken reward responses, and thereby promote disturbed eating behavior [30]. Consistent with this, smell and taste disorders in neurodegenerative disease are known to contribute to loss of appetite, reduced food intake, and weight loss [4]. For the GR, by contrast, only the right-sided volume was significantly lower in patients with eating disturbances, while the left and total volumes were not. This weaker, lateralized pattern may reflect hemispheric asymmetry in olfactory and frontal processing, but it should be interpreted with caution and confirmed in further studies.

Beyond these structural associations, OB volumetry showed strong diagnostic value. The total OB volume discriminated patients with AD from controls with excellent accuracy and high specificity, whereas the GR volumes showed lower but still clinically useful performance. Earlier work has likewise proposed OB and olfactory-tract volume as surrogate imaging markers of AD, with volumetric reductions detectable as early as the MCI stage [5,12,20]. These results suggest that OB volumetry, which can be obtained from routine MRI, may serve as a practical and accessible imaging marker to support the assessment of AD. Nevertheless, the conflicting negative findings noted above indicate that its diagnostic value, especially in early disease, still requires confirmation in prospective cohorts [11].

Gyrus rectus volume contributed diagnostic information beyond the olfactory bulb in distinguishing AD from controls, suggesting that frontal (gyrus rectus) atrophy is not merely a reflection of olfactory bulb loss but represents a partially independent component of AD-related neurodegeneration. This diagnostic contribution should be distinguished from the eating disturbance analysis, in which only olfactory bulb volume was independently associated, which indicates that the olfactory bulb, rather than the gyrus rectus, is the structure specifically linked to disturbed eating behavior.

### Limitations

Several limitations should be considered when interpreting these findings. The retrospective design did not allow standardized olfactory testing, such as the Sniffin’ Sticks or UPSIT, which would have enabled a more direct interpretation of functional olfactory impairment. Eating disturbances were identified from clinical records rather than from a structured assessment, which may have introduced misclassification. Several potential confounders of the association between brain volume and eating disturbances, including dementia severity, disease duration, nutritional status, depression, medication use, and intracranial volume, were not available in this cohort; the subgroup analyses could therefore be adjusted only for age and sex, and residual confounding cannot be excluded. In particular, the absence of intracranial volume precluded head size normalization, although the influence of global head size on structures as small as the olfactory bulb and gyrus rectus is likely modest. The subgroup findings should accordingly be regarded as hypothesis-generating rather than confirmatory. In addition, the ROC curves were derived and evaluated within the same sample, without an external validation cohort, cross-validation, or bootstrap resampling. Therefore, the reported AUC, sensitivity, and specificity values are likely optimistically biased and may overestimate performance in independent populations. The cross-sectional design also prevents any conclusion about causality. Finally, all measurements were performed by a single radiologist, and formal intra-rater reliability was not assessed. Nevertheless, the sample size was relatively large for an MRI-based study, a uniform imaging protocol was applied, and all measurements were carried out by a single experienced radiologist, which strengthens the consistency of the findings.

## 5. Conclusions

In conclusion, patients with AD showed substantial volume loss in both the OB and the GR compared with healthy controls. OB volume was closely associated with the presence of eating disturbances and discriminated patients with AD from controls with high accuracy, whereas GR involvement was also evident but followed a weaker and partly lateralized pattern in this selected cohort. These findings support a structural link between olfactory and frontal degeneration in AD and suggest that OB volumetry may have value as a promising imaging marker. These parameters should be interpreted strictly as preliminary exploratory findings rather than definitive evidence of clinical applicability, pending prospective validation in independent external cohorts. Prospective studies using standardized cognitive, olfactory, nutritional and imaging assessments are needed to determine clinical utility.

## Figures and Tables

**Table 1:** Demographic characteristics and eating disturbance status of the study groups.

Variable	Alzheimer’s Disease *n* = 135	Control *n* = 49	Total *n* = 184	*p*
**Age, Median [IQR]**	78 [71–81.5]	74 [71–80]	77 [71–81]	0.262
**Gender**	***n* (%)**	***n* (%)**	***n* (%)**	0.907
Female	94 (69.6)	33 (67.3)	127 (69.0)	
Male	41 (30.4)	16 (32.7)	57 (31.0)	
**Eating disturbances**				<0.001
No	47 (34.8)	49 (100.0)	96 (52.2)	
Yes	88 (65.2)	0 (0.0)	88 (47.8)	

Continuous variables are presented as median [Q1–Q3]. Categorical variables are presented as *n* (%). Age was compared using the Mann–Whitney U test. Gender was compared using the chi-square test. Eating disturbances were compared using Fisher’s exact test.

**Table 2:** Comparison of olfactory bulb and gyrus rectus volumes between patients with Alzheimer’s disease and controls, with effect sizes and adjusted associations.

Variable	AD, Median [IQR]	Control, Median [IQR]	W	Raw *p*	FDR-Adjusted *p*	Effect Size, *r* (95% CI) ^a^	Adjusted OR (95% CI) ^b^
Right OB volume	0.041 [0.031–0.051]	0.072 [0.065–0.077]	374.0	<0.001	<0.001	0.89 (0.84–0.92)	0.089 (0.051–0.197)
Left OB volume	0.037 [0.028–0.050]	0.064 [0.060–0.071]	506.5	<0.001	<0.001	0.85 (0.78–0.89)	0.127 (0.061–0.240)
Total OB volume	0.079 [0.060–0.102]	0.139 [0.126–0.146]	349.0	<0.001	<0.001	0.89 (0.85–0.93)	0.083 (0.049–0.178)
Right GR volume	1.660 [1.255–2.230]	2.600 [1.900–3.236]	1470.5	<0.001	<0.001	0.56 (0.41–0.67)	0.265 (0.163–0.406)
Left GR volume	1.650 [1.190–2.065]	2.796 [1.980–3.250]	1057.5	<0.001	<0.001	0.68 (0.57–0.77)	0.184 (0.105–0.298)
Total GR volume	3.400 [2.560–4.110]	5.482 [4.030–6.680]	1170.0	<0.001	<0.001	0.65 (0.52–0.74)	0.196 (0.113–0.315)

Note: Data are presented as median [interquartile range]. Between-group comparisons were performed using the Mann–Whitney U test. Raw *p*-values were adjusted for six volumetric comparisons using the Benjamini–Hochberg false discovery rate procedure. OB, olfactory bulb; GR, gyrus rectus; OR, odds ratio; CI, confidence interval. ^a^ Effect size r and corresponding 95% confidence intervals were calculated for the Mann–Whitney U comparisons. ^b^ Adjusted odds ratios were obtained from separate multivariable logistic regression models using standardized volumetric measurements.

**Table 3:** Comparison of olfactory bulb and gyrus rectus volumes according to eating disturbances status in patients with Alzheimer’s disease, with effect sizes and adjusted associations.

Variable	No Eating Disturbances, Median [IQR]	Eating Disturbances, Median [IQR]	W	Raw *p*	FDR-Adjusted *p*	Effect Size, *r* (95% CI) ^a^	Adjusted OR (95% CI) ^b^
Right OB volume	0.057 [0.047–0.066]	0.036 [0.028–0.041]	3696.5	<0.001	<0.001	0.79 (0.70–0.85)	0.064 (0.020–0.164)
Left OB volume	0.051 [0.043–0.054]	0.033 [0.025–0.038]	3565.5	<0.001	<0.001	0.72 (0.61–0.81)	0.130 (0.050–0.283)
Total OB volume	0.106 [0.093–0.121]	0.066 [0.056–0.079]	3713.5	<0.001	<0.001	0.80 (0.71–0.86)	0.072 (0.023–0.178)
Right GR volume	1.800 [1.560–2.300]	1.650 [1.245–2.125]	2517.0	0.038	0.057	0.22 (0.02–0.40)	0.797 (0.445–1.440)
Left GR volume	1.820 [1.310–2.100]	1.640 [1.120–1.992]	2335.0	0.218	0.218	0.13 (−0.08–0.32)	0.891 (0.495–1.610)
Total GR volume	3.620 [2.745–4.230]	3.150 [2.470–4.100]	2463.0	0.068	0.082	0.19 (−0.01–0.38)	0.818 (0.439–1.520)

Note: Data are presented as median [interquartile range]. Between-group comparisons were performed using the Mann–Whitney U test. Raw *p*-values were adjusted for six volumetric comparisons using the Benjamini–Hochberg false discovery rate procedure. OB, olfactory bulb; GR, gyrus rectus; OR, odds ratio; CI, confidence interval. ^a^ Effect size r and corresponding 95% confidence intervals were calculated for the Mann–Whitney U comparisons. ^b^ Adjusted odds ratios were obtained from separate multivariable logistic regression models using standardized volumetric measurements.

**Table 4:** Age- and gender-adjusted logistic regression analysis for Alzheimer’s disease.

Variable	Adjusted OR 95% CI	*p*	VIF	Tolerance
Right olfactory bulb volume	0.089 (0.051–0.197)	<0.001	1.191	0.840
Left olfactory bulb volume	0.127 (0.061–0.240)	<0.001	1.130	0.885
Total olfactory bulb volume	0.083 (0.049–0.178)	<0.001	1.183	0.846
Right gyrus rectus volume	0.265 (0.163–0.406)	<0.001	1.088	0.919
Left gyrus rectus volume	0.184 (0.105–0.298)	<0.001	1.048	0.954
Total gyrus rectus volume	0.196 (0.113–0.315)	<0.001	1.064	0.939

Logistic regression analyses were performed with Alzheimer’s disease status as the dependent variable. Each MRI volumetric variable was entered into a separate model and adjusted for age and gender. MRI volumetric variables were standardized before analysis; therefore, odds ratios represent the change in odds of Alzheimer’s disease per 1-standard-deviation increase in the corresponding MRI volumetric measurement. VIF and tolerance values were calculated to assess multicollinearity.

**Table 5:** Comparison of nested multivariable logistic regression models for discriminating patients with Alzheimer’s disease from controls.

Model	Predictors	Adjusted OR (95% CI) ^a^	AUC (95% CI)	AIC	LRT vs. Previous Model	DeLong *p* vs. Previous Model
1 (base)	Age + sex	—	0.554 (0.463–0.645)	218.9	—	—
2 (+OB)	Age + sex + total OB volume	OB: 0.011 (0.002–0.043)	0.963 (0.939–0.987)	86.3	<0.001	<0.001
3 (+GR)	Age + sex + total OB volume + total GR volume	OB: 0.011 (0.001–0.052); GR: 0.284 (0.119–0.579)	0.977 (0.960–0.995)	74.8	<0.001	0.039

Note: OB, olfactory bulb; GR, gyrus rectus; OR, odds ratio; CI, confidence interval; AUC, area under the receiver operating characteristic curve; AIC, Akaike information criterion; LRT, likelihood-ratio test. LRT and DeLong *p*-values compare each model with the immediately preceding model. Lower AIC values indicate better model fit. ^a^ Odds ratios for volumetric variables are reported per 1-standard-deviation increase.

## Data Availability

The original contributions presented in this study are included in the article. Further inquiries can be directed to the corresponding author.

## References

[1] Abubakar M.B., Sanusi K.O., Ugusman A., Mohamed W., Kamal H., Ibrahim N.H., Khoo C.S., Kumar J. (2022). Alzheimer’s disease: An update and insights into pathophysiology. Front. Aging Neurosci..

[2] Nandi A., Counts N., Chen S., Seligman B., Tortorice D., Vigo D., Bloom D.E. (2022). Global and regional projections of the economic burden of Alzheimer’s disease and related dementias from 2019 to 2050: A value of statistical life approach. eClinicalMedicine.

[3] Selles M.C., Oliveira M.M., Ferreira S.T. (2018). Brain inflammation connects cognitive and non-cognitive symptoms in Alzheimer’s disease. J. Alzheimer’s Dis..

[4] DeVere R. (2017). Disorders of taste and smell. Continuum.

[5] Jobin B., Boller B., Frasnelli J. (2021). Volumetry of olfactory structures in mild cognitive impairment and Alzheimer’s disease: A systematic review and a meta-analysis. Brain Sci..

[6] Fatuzzo I., Niccolini G.F., Zoccali F., Cavalcanti L., Bellizzi M.G., Riccardi G., de Vincentiis M., Fiore M., Petrella C., Minni A. (2023). Neurons, nose, and neurodegenerative diseases: Olfactory function and cognitive impairment. Int. J. Mol. Sci..

[7] Herrmann T., Koeppel C., Linn J., Croy I., Hummel T. (2023). Olfactory brain activations in patients with major depressive disorder. Sci. Rep..

[8] Ide S., Murakami Y., Futatsuya K., Anai K., Yoshimatsu Y., Fukumitsu S., Tsukamoto J., Hashimoto T., Adachi H., Ueda I. (2024). Usefulness of olfactory bulb measurement in 3D-FIESTA in differentiating Parkinson disease from atypical parkinsonism. Am. J. Neuroradiol..

[9] Simonini L., Frijia F., Ait Ali L., Foffa I., Vecoli C., De Gori C., De Cori S., Baroni M., Aquaro G.D., Maremmani C. (2024). A comprehensive review of COVID-19-related olfactory deficiency: Unraveling associations with neurocognitive disorders and magnetic resonance imaging findings. Diagnostics.

[10] Naffaa M.M. (2025). Neurogenesis dynamics in the olfactory bulb: Deciphering circuitry organization, function, and adaptive plasticity. Neural Regen. Res..

[11] Servello A., Fioretti A., Gualdi G., Di Biasi C., Pittalis A., Sollaku S., Pavaci S., Tortorella F., Fusetti M., Valenti M. (2015). Olfactory dysfunction, olfactory bulb volume and Alzheimer’s disease: Is there a correlation? A pilot study. J. Alzheimer’s Dis..

[12] Thomann P.A., Dos Santos V., Seidl U., Toro P., Essig M., Schröder J. (2009). MRI-derived atrophy of the olfactory bulb and tract in mild cognitive impairment and Alzheimer’s disease. J. Alzheimer’s Dis..

[13] Bothwell A.R., Resnick S.M., Ferrucci L., Tian Q. (2023). Associations of olfactory function with brain structural and functional outcomes. A systematic review. Ageing Res. Rev..

[14] Gray J.P., Müller V.I., Eickhoff S.B., Fox P.T. (2020). Multimodal abnormalities of brain structure and function in major depressive disorder: A meta-analysis of neuroimaging studies. Am. J. Psychiatry.

[15] Abubakar M., Giri A., Goel F., Khan M., Gupta J., Kumar D., Kaushik M., Rai S.N., Kumar N. (2026). Diabetes, Alzheimer’s disease risk factors, and the cafeteria diet: A comprehensive review. Curr. Neuropharmacol..

[16] Stark R. (2024). The olfactory bulb: A neuroendocrine spotlight on feeding and metabolism. J. Neuroendocrinol..

[17] Torres-Pasillas G., Chi-Castañeda D., Carrillo-Castilla P., Marín G., Hernández-Aguilar M.E., Aranda-Abreu G.E., Manzo J., García L.I. (2023). Olfactory dysfunction in Parkinson’s disease, its functional and neuroanatomical correlates. NeuroSci.

[18] Zhang B., Rolls E.T., Wang X., Xie C., Cheng W., Feng J. (2024). Roles of the medial and lateral orbitofrontal cortex in major depression and its treatment. Mol. Psychiatry.

[19] Hummel T., Guerra N.P., Gunder N., Hähner A., Menzel S. (2023). Olfactory function and olfactory disorders. Laryngorhinootologie.

[20] Thomann P.A., Dos Santos V., Toro P., Schönknecht P., Essig M., Schröder J. (2009). Reduced olfactory bulb and tract volume in early Alzheimer’s disease—A MRI study. Neurobiol. Aging.

[21] Gupta K. (2025). Introduction to Alzheimer’s disease and biomarkers. Deep Generative Models for Integrative Analysis of Alzheimer’s Biomarkers.

[22] Carnemolla S.E., Kumfor F., Liang C.T., Foxe D., Ahmed R.M., Piguet O. (2022). Olfactory bulb integrity in frontotemporal dementia and Alzheimer’s disease. J. Alzheimer’s Dis..

[23] Jacobson S., Pugsley S., Marcus E.M. (2025). The limbic system: Temporal lobe, prefrontal cortex, and learning, memory, and emotions. Neuroanatomy for the Neuroscientist.

[24] Han K.M., Ham B.J. (2021). How inflammation affects the brain in depression: A review of functional and structural MRI studies. J. Clin. Neurol..

[25] Frings L., Yew B., Flanagan E., Lam B.Y., Hüll M., Huppertz H.J., Hodges J.R., Hornberger M. (2014). Longitudinal grey and white matter changes in frontotemporal dementia and Alzheimer’s disease. PLoS ONE.

[26] Ramirez M.K., Phipps C.J., Murman D.L., Beadle J.N., Phatak V.S., Warren D.E. (2025). Structural neuroimaging correlates of neuropsychiatric symptoms in Alzheimer’s disease: A systematic literature review. Dement. Geriatr. Cogn. Disord..

[27] Lindberg O., Westman E., Karlsson S., Östberg P., Svensson L.A., Simmons A., Wahlund L.-O. (2012). Is the subcallosal medial prefrontal cortex a common site of atrophy in Alzheimer’s disease and frontotemporal lobar degeneration?. Front. Aging Neurosci..

[28] Shepherd G.M., Rowe T.B., Greer C.A. (2021). An evolutionary microcircuit approach to the neural basis of high dimensional sensory processing in olfaction. Front. Cell. Neurosci..

[29] Valotassiou V., Sifakis N., Tzavara C., Lykou E., Tsinia N., Kamtsadeli V., Sali D., Angelidis G., Psimadas D., Tsougos I. (2021). Eating disorders in frontotemporal dementia and Alzheimer’s disease: Evaluation of brain perfusion correlates using 99mTc-HMPAO SPECT with Brodmann areas analysis. J. Alzheimer’s Dis..

[30] Godyla-Jabłoński M., Pachura N., Klemens M., Wolska J., Łyczko J. (2025). Natural appetite control: Food-derived aromas as appetite decreasing agents—A proof-of-concept study. Nutrients.

